# Immunogold Nanoparticles for Rapid Plasmonic Detection of *C. sakazakii*

**DOI:** 10.3390/s18072028

**Published:** 2018-06-25

**Authors:** Mohamed A. Aly, Konrad J. Domig, Wolfgang Kneifel, Erik Reimhult

**Affiliations:** 1 Department of Nanobiotechnology, Institute for Biologically Inspired Materials, BOKU—University of Natural Resources and Life Sciences, Vienna, Vienna A-1190, Austria; mohamed.aly@boku.ac.at; 2 Department of Food Science, Faculty of Agriculture, Ain Shams University, P.O. Box 68, Hadayek Shobra, Cairo 1124, Egypt; 3 Department of Food Science and Technology, Institute of Food Science, BOKU—University of Natural Resources and Life Sciences, Vienna, Vienna A-1190, Austria; konrad.domig@boku.ac.at (K.J.D.); wolfgang.kneifel@boku.ac.at (W.K.)

**Keywords:** gold nanoparticles, targeted bacteria, antibody labeling, *Cronobacter sakazakii*, immunogold, plasmon extinction nanoparticle sensing, poly(ethylene glycol) brush, spectroscopic detection

## Abstract

*Cronobacter sakazakii* is a foodborne pathogen that can cause a rare, septicemia, life-threatening meningitis, and necrotizing enterocolitis in infants. In general, standard methods for pathogen detection rely on culture, plating, colony counting and polymerase chain reaction DNA-sequencing for identification, which are time, equipment and skill demanding. Recently, nanoparticle- and surface-based immunoassays have increasingly been explored for pathogen detection. We investigate the functionalization of gold nanoparticles optimized for irreversible and specific binding to *C. sakazakii* and their use for spectroscopic detection of the pathogen. We demonstrate how 40-nm gold nanoparticles grafted with a poly(ethylene glycol) brush and functionalized with polyclonal antibodies raised against *C. sakazakii* can be used to specifically target *C. sakazakii*. The strong extinction peak of the Au nanoparticle plasmon polariton resonance in the optical range is used as a label for detection of the pathogens. Individual binding of the nanoparticles to the *C. sakazakii* surface is also verified by transmission electron microscopy. We show that a high degree of surface functionalization with anti-*C. sakazakii* optimizes the detection and leads to a detection limit as low as 10 CFU/mL within 2 h using a simple cuvette-based UV-Vis spectrometric readout that has great potential for further optimization.

## 1. Introduction

*Cronobacter sakazakii* (*C. sakazakii*) is an emerging opportunistic foodborne pathogen that can infect infants and neonates resulting in meningitis, necrotizing enterocolitis, and bacteremia, with a 40–80% mortality rate [1,2]. Methods to detect foodborne pathogens as potential biological threats to our health and economy have increasingly become a priority [3]. Detection of bacterial pathogens is a crucial issue for effective clinical diagnosis and treatment. Even more important would be early detection in production facilities and critical environments to ensure the safety of the food supply so that infections cannot take place [4]. Traditional cell plating and colony counting analysis methods provide reliable results for identification and detection of pathogens in food, but they are very time consuming [5], i.e., they require more than one day to deliver results. For example, the standard protocol for testing the presence of *C. sakazakii* suggested by the Federal Drug Administration requires 2–3 days [6]. Such tests are not suitable for rapid and continuous monitoring and also not useful to determine timing of cleaning cycles or other interventions in an industrial or clinical setting. Sensitive detection of *C. sakazakii* has also been performed using, e.g., real-time quantitative PCR and reached detection limit <10^3^ CFU/mL [7], but this method is not generally recommended since it requires an expensive thermal cycler with a fluorescence detector [8].

Immunological methods are today frequently developed for the detection and identification of foodborne pathogens; they require less assay time than traditional culture techniques and have high specificity toward the target pathogen [9]. Immunological methods include the enzyme-linked immunosorbent assay (ELISA), which is a sensitive, specific and convenient method to detect macromolecules, proteins and bacteria [10,11]. Sandwich ELISA and indirect non-competitive ELISA for the detection of *Cronobacter muytjensii* have reached a detection limit of 2.0 × 10^4^ CFU/mL (colony forming units per milliliter) in pure culture [12,13]. In comparison, an indirect non-competitive ELISA using a sandwich assay of monoclonal antibodies was capable of detecting ~10^5^ CFU/mL of *C. sakazakii* with a linear range as function of the logarithm of concentration between 10^5^ and 10^8^ CFU/mL [14]. This ELISA could be performed within one day in approximately 8 h. In a recent study using a fluorescently tagged liposomes functionalized with antibodies, rapid detection of *C. muytjensii* with a detection limit of 6.3 × 10^4^ CFU/mL was achieved in pure culture [15].

To increase sensitivity and to achieve other advantages such as enhanced extraction of bacteria to facilitate readout, research has increasingly turned to the use of targeted nanoparticles, in particular immunolabeled nanoparticles. Such “immunogold” particles have long been a staining technology for biochemical identification in electron microscopy, since they in principle allow for imaging of the distribution of biomolecules on e.g., cell membranes [16,17]. The advantages of nanoparticle-based antibody labels, such as high optical (plasmonic extinction and surface-enhanced Raman scattering) signals, magnetic properties, increased affinity, more rapid and efficient binding than surface-based methods and high electron density contrast for electron microscopy imaging, have made them increasingly attractive to be used as biochemical sensors, nanoscale building blocks and immunohistochemical probes [18,19,20,21]. Several novel detection schemes have been proposed that utilize the special properties of the nanometric inorganic particles. In a recent study, gold nanoparticles (NPs) functionalized directly with antibodies used the proximity of the gold NP cores to accelerate the electron transfer rate and consequently amplify the detection signal of the nanoprobe in an electrochemical assay [22]. Antibody functionalized gold NPs have also been used for the detection of *Escherichia coli* O157:H7 by using inductively coupled plasma mass spectrometry; this highly sensitive assay was able to detect as few as 500 CFU/mL [23], but requires advanced equipment and preparation. Another study demonstrated that ferromagnetic nanoparticles with a size of 30 nm functionalized with anti–*Listeria monocytogenes* antibodies via streptavidin-biotin bonds could be used not only to detect but also to capture *Listeria monocytogenes* in a pure sample during a 2 h immunoreaction [24]. Wang and Alocilja recently demonstrated a rapid and sensitive method for bacterial detection reaching a sensitivity of 10 CFU/mL with a linear range of 10^1^–10^6^ CFU/mL for *E. coli* O157:H7 [25]. They used polymer-coated magnetic nanoparticles to first extract *E. coli* O157:H and then performed a sandwich assay for electrochemical readout of Au nanoparticles targeted to the extracted *E. coli*, which could be completed within 1 h. Ngo et al. (2015) also attempted labeling for transmission electron microscopy, UV-Vis spectroscopy and FTIR of *E. coli* O157:H7 with Au nanoparticles (12–14 nm diameter) stabilized by thiol surfactant (3-mercaptopropionic) and directly conjugated with anti-*E. coli* O157:H7 antibodies [26]. An additional report on the detection of *E. coli* 0157:H7 in urine sample used Au NPs conjugated with cysteine and unspecific electrostatic adhesion to *E. coli* [27]. This method showed a linear relationship in the range of 1 × 10^3^–4 × 10^3^ cells/mL and claimed a detection limit of 100 cells/mL, plus the possibility to detect *E. coli* with the naked eye. Gold-coated magnetic nanoparticle clusters (Au/MNCs) functionalized with antibodies were used to detect *Salmonella* in milk sample; the limit of detection for *Salmonella* using these clusters was demonstrated as 10^3^ CFU/mL [28].

Nanoparticle-based assays have also been applied to detection of *C. sakazakii*. Rapid, sensitive and efficient detection of *C. sakazakii* was achieved using immunoliposomes containing magnetic nanoparticles and readout of fluorophores encapsulated in the liposomes after magnetic separation [8]. This approach could detect <10^4^ CFU/mL within 2.5 h and the measurements had a linear range up to 10^6^ CFU/mL as a function of the logarithm of concentration. In another recent report, a lateral flow immunoassay (LFA) was developed for equally sensitive detection of *C. sakazakii*, which took advantage of an immunogold sandwich assay to improve sensitivity [18]. Using gold nanoparticles functionalized with monoclonal antibodies, the LFA strip test could be made highly specific to *C. sakazakii* with a detection limit of almost 10^3^ CFU/mL (two orders of magnitude more sensitive than standard LFA) and a detection time shortened by 3 h compared to other LFA.

Although recent progress has been strong, most of these techniques suffer from limitations such as time consuming multiple steps for enrichment and plating, expensive and non-portable equipment, as well as specialized training required for operation [29]. Additionally, lower detection limits are still desired. Hence, exploring sensitive, specific and rapid analytical methods to detect pathogens such as *C. sakazakii* remains an important task. New analytical methods for rapid detection techniques should ideally also be more user-friendly, require simpler and cheaper equipment, less technical expertise and be more time efficient compared to the advanced analytical techniques currently in use.

Successful biosensors require low limits of detection. Achieving this depends on using a high amplification sensor scheme and reaching high target specificity and low background signal. The latter two require, especially for nanoscale sensor elements, appropriate and stable molecular surface modification [30]. A gold nanoparticle core provided the high signal for many of the novel readout formats presented above. However, the NP surface must be properly functionalized to achieve the required specific targeting. To protect the nanoparticles from nonspecific binding to proteins and cell surfaces it is proper to modify the surface with a hydrophilic polymer brush anchored to the surface by a thiol [31,32]. Specific and selective binding is introduced by functionalizing the polymer brush with a high-affinity ligand for the target. Antibodies provide the necessary specificity in such applications and can be chemically or biochemically coupled to the nanoparticle surface [33,34], as demonstrated by the many examples given above. An optical readout can be highly sensitive when a nanoparticle marker is used for enhancement. Tentatively, sensitivities can be reached such that in-line detection of single events using droplet or microfluidic channel-based readout formats are possible. In any case, monodisperse gold nanoparticles with designed plasmonic resonances properly functionalized with antibodies allow for fast (sub-second to a minute depending on platform) readout of the presence of immunolabels using extinction spectroscopy.

**Figure 1:**
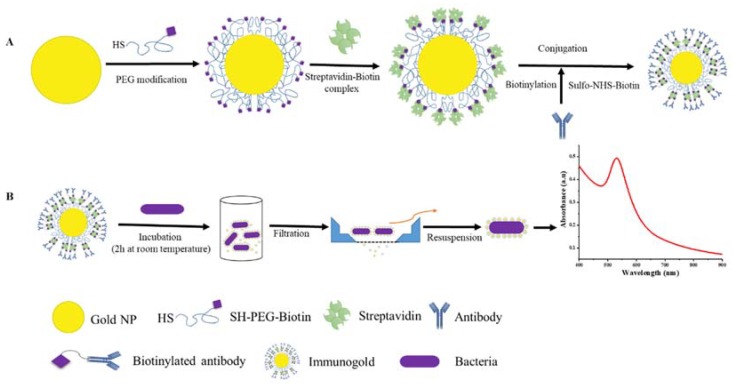
(**A**) Schematic of the functionalization route for PEG-grafted gold nanoparticles and conjugation with anti-*C. sakazakii* IgG via streptavidin-biotin coupling; (**B**) the resulting immunogold nanoparticles specifically bind to *C. sakazakii* after incubation. A fast and simple centrifugation and filtration step makes the specifically labeled bacteria detectable with plasmon extinction spectroscopy.

To shorten the detection time and simplify the detection procedure while obtaining a very low detection limit we therefore investigate gold nanoparticles functionalized with antibodies for detection of *C. sakazakii* in a simple two-step spectroscopic assay. This study demonstrates functionalization of poly(ethylene glycol) (PEG) brush-stabilized gold NPs with polyclonal antibodies for specific binding to *C. sakazakii* and the proof of concept of using these targeted nanoparticles in a simple platform for *C. sakazakii* detection using extinction spectroscopy (Figure 1). Gold nanoparticles with a diameter of ~40 nm were synthesized via citrate reduction of gold salts following the Turkevich method to have a suitable platform for PEG and antibody functionalization and an easily measurable optical extinction spectrum in the visible range. The gold nanoparticles were stabilized with a polymer brush to suppress undesired non-specific binding using thiol-PEG with a biotin end-terminal group. The fraction of biotin end-groups can be varied to control the density of biotinylated affinity ligands bound to the nanoparticle surface using a streptavidin linker. We use polyclonal biotinylated rabbit antibodies raised against *C. sakazakii* (anti-*C. sakazakii*) anchored to the streptavidin-functionalized nanoparticle surface. Figure 1 schematically shows the step-by-step build-up of the Au NPs functionalized with antibodies for specific targeting of *C. sakazakii*. The optical detection of *C. sakazakii* using the antibody-gold NPs was demonstrated using extinction spectroscopy and with specific binding of the nanoparticles on the cell surface verified by transmission electron microscopy.

## 2. Materials and Methods

### 2.1. Materials and Reagents

Hydrogen tetrachloroaurate (III) hydrate (HAuCl_4_), Biotin 3-sulfo-*N*-hydroxysuccinimide ester sodium salt, AgNO_3_, ascorbic acid, sodium citrate, streptavidin and phosphate buffered saline (10× PBS) were purchased from Sigma-Aldrich (St. Louis, MI, USA). The α-hydroxy-ω-mercapto-poly(ethylene glycol) (MW 5 kDa) (mPEG-SH) was purchased from Jenkem Technology (Plano, TX, USA). α-hydroxy-ω-biotin-poly(ethylene glycol) (MW 5 kDa) (HS-PEG-Biotin was purchased from RAPP Polymer, Tübingen, Germany. IgG from rabbit serum was purchased from Sigma and tryptone soya broth from Oxoid.

### 2.2. Bacteria Culture and Plate Counting

C. sakazakii DSM 4485, Escherichia coli DSM 613, Cronobacter turicensis DSM 18703, Cronobacter dublinensis dublinensis DSM 18705, Cronobacter muytjensii ATCC 51329, Cronobacter universalis DSM 27963, Cronobacter condimenti DSM 27966, Cronobacter malonaticus DSM 18702, Salmonella Typhimurium DSM 554, Salmonella enterica subsp. enterica DSM 14221, Enterobacter xiangfangensis LMG 27195 and Staphylococcus aureus subsp. aureus DSM 799 were grown in tryptic soy broth (TSB) at 37 °C overnight. To count the number of bacteria, cultures were serially diluted with sterile 0.85% NaCl, and 100 μL of the selected dilution was then cultured on tryptic soy agar (TSA) spread plates in triplicates followed by incubation at 37 °C for 24 h.

### 2.3. Antibody Production and Measurement of Coupled Antibody by Western Blot

A rabbit polyclonal antibody against the whole bacteria cell of *C. sakazakii* was produced (see Supplementary Materials for details on all steps of antibody production and functionalization), and anti-*C. sakazakii* IgG was purified by a two-step purification method using caprylic acid and ammonium sulfate precipitate as previously described [35]. The purity of prepared rabbit anti-*C. sakazakii* IgG was checked by sodium dodecyl sulfate-polyacrylamide gel electrophoresis (SDS-PAGE) (Supplementary Materials). Western blot was also performed with a primary antibody of anti-*C. sakazakii* that recognizes proteins on the bacteria cell surface and the results are available in the Supplementary Materials.

### 2.4. Biotin Conjugated to Anti–C. sakazakii IgG

Polyclonal rabbit anti–*C. sakazakii* antibodies (IgG) at a concentration of 2.5 mg/mL were prepared as described above immediately before use. 10 mM biotin 3-sulfo-*N*-hydroxysuccinimide ester sodium salt solution was prepared by dissolving 2.2 mg of biotin 3-sulfo-*N*-hydroxysuccinimide ester sodium salt (Sigma-Aldrich) in 0.5 mL of milli-Q water. 10 µL of prepared (at a 30-fold molar excess over IgG) biotin 3-sulfo-*N*-hydroxysuccinimide (10 mM) solution and 200 µL of rabbit anti–*C. sakazakii* IgG at original concentration was added to 400 µL of PBS*.* The mixture was incubated for 1 h at room temperature and then overnight at 4 °C. The buffer exchange to remove uncoupled biotin was performed using a dialysis-membrane cut-off of 12–16 kDa pore size 25A (Biomol GmbH, Hamburg, Germany). The membrane was immersed in PBS and dialyzed at room temperature for 2 h. PBS was removed and changed, and the mixture was dialyzed for another 2 h at room temperature. Later, PBS was changed one more time, then dialyzed overnight at 4 °C. Biotin-conjugated anti–*C. sakazakii* IgG antibodies were stored at −20 °C until use.

### 2.5. Gold Nanoparticle Synthesis and Functionalization

Gold NPs with a diameter of 40 nm were prepared by seed-mediated synthesis [36]. The Supplementary Materials contain detailed information on all preparation steps to functionalize the nanoparticles. In brief, to vary the surface density of biotin on the outer surface of poly(ethylene glycol)-brush stabilized gold NPs, they were modified with mixtures of mPEG-SH and HS-PEG-Biotin. HS-PEG-Biotin and mPEG-SH were prepared fresh by dissolving in milli-Q water to a concentration of 3 mM to obtain molar fractions of 0, 5, 10, 20, 40, 50–100% HS-PEG-Biotin/mPEG-SH. Gold NPs were incubated with the respective PEG-thiol mixture under thorough mixing by gentle swirling overnight at 4 °C. The gold particles were purified from excess PEG-thiol and purified by seven repeated sequentl washing steps in Milli-Q water using Amicon Ultra centrifugal ultra-4 filter with 100 kDa cut-off (Merck Millipore, Ltd., Tullagreen, Ireland). The centrifugation was performed at 800× *g* for 10 min at 4 °C followed by resuspension in Milli-Q water. This yielded colloidally stable particles shielded by a PEG-brush as shown previously [37]. The nanoparticles were diluted in NaCl (10 mM, pH 7.2) solution. Different samples were incubated at room temperature for 5 min and then analyzed; the hydrodynamic diameters and Zeta-potentials for the gold NPs were acquired by dynamic light scattering (DLS) using a Malvern Zeta Sizer, NanoZS (Malvern Instrument Ltd, Malvern, UK) instrument. The size distribution of the gold NPs was determined from transmission electron micrographs (TEM, see below for the description of TEM sample preparation and measurement) using the public domain image processing software ImageJ and its tool to analyze particles. The average gold NP size was calculated by fitting a Gaussian distribution to the size distributions obtained by the TEM analysis. Thermal gravimetric analysis of the nanoparticles was performed on a Mettler Toledo TGA/DSC 1 (Mettler Toledo GmbH, Vienna, Austria), with 80 mL min^−1^ synthetic air as reactive gas, 20 mL min^−1^ nitrogen as protective gas and a heating rate of 10 K min^−1^ from 25 to 650 °C. Using TGA results, the grafting density (σ) was calculated using the following formula:

$$\sigma =\frac{{\left(\%\;w/w\right)}_{shell}\;\rho_{Au}V_{core}\;N_{A}}{{\left(\%\;w/w\right)}_{core}\;M_{PEG\;}A_{core}}$$

where ${\left(\%\;w/w\right)}_{shell}$ is percentage of mass loss in TGA for the organic fraction corresponding to the PEG grafted to the gold core; $N_{A}$ is the Avogadro constant; $\rho_{Au}$ is the density of gold; $V_{core}$ is the volume; $A_{core}$ is the area of the gold core, calculated from the diameter of the cores measured by TEM; $M_{PEG}$ is the molecular weight of the PEG; and ${\left(\%\;w/w\right)}_{core}$. is the residual mass percentage of the inorganic fraction in TGA.

### 2.6. Streptavidin-Biotin Coupling

The gold NPs modified by 0, 5, 10, 20, 50–100% HS-PEG-Biotin/mPEG-SH were incubated 2 h at room temperature or extended to overnight incubation at 4 °C. The streptavidin was added by slow, drop-wise addition of 100 µL gold NP solution (1 nM) to 500 µL solution of streptavidin (1.5 µM) in Binding Buffer (1× PBS, pH 7.4). After incubation, the gold NPs were separated from unbound streptavidin by centrifuging at 800× *g* for 15 min at 4 °C using Amicon Ultra centrifugal ultra-4 filter with a 100 kDa cut-off. The supernatant was removed, then the particles were washed three times in Milli-Q water to remove the excess streptavidin. Streptavidin-functionalized gold NPs were resuspended in PBS. This suspension was vortexed and agitated for 2 h at 4 °C.

### 2.7. Preparation of Immunogold Nanoparticles

50 uL of gold NPs were added to 100 uL of PBS and mixed with 100 µL of biotin-conjugated anti-*C. sakazakii* IgG (0.8 mg/mL) and rotated continuously for 2 h at 30 rpm on a shaker (KS 501 digital, Ika-Werke, Staufen, Germany) at room temperature. Then, the pellet was washed two times with PBS to remove unbound antibodies. The gold NPs coated with IgG were resuspended in 100 µL of PBS. Immunogold NPs were stored at 4 °C. Nanoparticles were used within weeks for the reported experiments but are stable and functional over at least 6 months of storage in solution.

### 2.8. Preparation of C. sakazakii Targeted by Immunogold Nanoparticles

The serial dilutions of *C. sakazakii* ranging from 10^1^ to 10^7^ CFU/mL were prepared in physiological saline, and 100 μL of each dilution was added to 100 µL of immunogold NPs. This mixture was incubated at room temperature for 2 h in a shaking incubator at 30 rpm. The same number of bacteria were also incubated with PEG-grafted Au nanoparticles as a negative control. The filter membrane with 100 nm diameter pores was wetted in physiological saline for 3 min before using. Subsequently, 200 μL of the resuspended bacteria-immunogold NPs complexes were filtered through the filter membrane using pressure injection by hand. While the unbound gold NPs, which have much smaller diameter than the membrane pores, could pass through the filter membrane, immunogold NPs bound to *C. sakazakii* form too large complexes to penetrate through the pores and remained on the membrane surface. This step was followed by a one-time washing using 200 µL physiological saline to remove residual free or weakly bound gold NPs. This procedure takes ~10 min/sample. The immunogold-labeled bacteria cells captured on the filter membrane surface were recovered by injection of physiological saline from the opposite direction. This step was repeated three times, followed by centrifugation and resuspension in 200 µL physiological saline and then used for measurements.

### 2.9. Extinction Spectroscopy

Bacterial samples and samples of Au nanoparticles and combinations of the two were characterized using UV–Vis spectroscopy (Hitachi, Tokyo, Japan, model: U-2900). Extinction spectra were recorded using disposable (cuvettes pathlength 10 mm, chamber volume 1600 μL) for wavelengths ranging from 400 to 900 nm. All experiments were performed at least in triplicates to check for reproducibility and resulting in standard deviations of ~10%.

### 2.10. Transmission Electron Microscopy

Particle size, uniformity, and shape were measured using transmission electron microscopy (TEM), studies were performed on a FEI Tecnai G2 20 transmission electron microscope operating at 160 kV. A droplet of the nanoparticle dispersion was placed on a carbon-coated copper grid. The excess of the solution on the grating was blotted away after 30 s by filter paper and then dried. TEM was also performed on the bacteria after incubation with the targeted Au nanoparticles and separation of unbound nanoparticles. 10 µL of the resulting dispersion was placed as a droplet on parafilm and then brought in contact with a carbon-coated grid for 2 min. The grid was then transferred and put in contact with 10 uL of 2.5% glutaraldehyde in cacodylate buffer placed on parafilm for 10 min for fixation, followed by washing 3 times. Each step was followed by removal of excess by using a filter. Finally, the sample was dried in air and transferred to the TEM for imaging.

## 3. Results and Discussion

The polyclonal rabbit anti-*C. sakazakii* antibodies (immunoglobulin G (IgG)) were produced, and their purity examined by SDS-PAGE (Figure S1). The results confirm that the rabbit anti-*C. sakazakii* IgG has high purity, comparable to commercial rabbit IgG and, hence, is suitable for use in an immunoassay for the detection of *C. sakazakii*. The specificity of anti-*C. sakazakii* antibody was determined with Western blot on whole bacterial cells for *C. sakazakii* and related species. The Western blot reported in Figure S2 and in Table S1 shows high binding activity to *C. sakazakii* while no binding was observed to any of the other tested *Cronobacter*, *Enterobacter*, *Escherichia*, *Salmonella* or *Staphylococcus* species.

**Figure 2:**
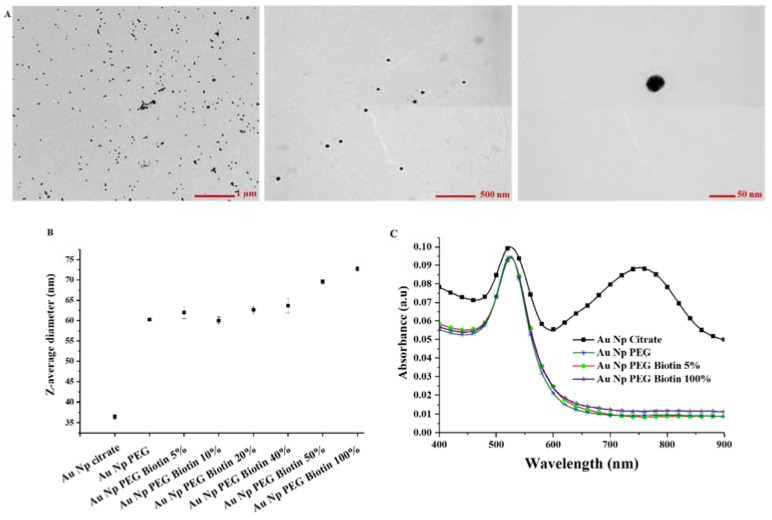
Characterization of as-synthesized and PEG-grafted gold nanoparticles. (**A**) Transmission electron micrographs of as-synthesized and citrate stabilized 40-nm gold nanoparticles; (**B**) hydrodynamic size of gold nanoparticles measured by dynamic light scattering. The nanoparticles are functionalized by a PEG polymer brush containing different fractions of PEG with biotin end-group functionality; (**C**) UV-Vis extinction spectra for as-synthesized and PEG-grafted Au NPs showing deaggregation in physiological saline (145.4 M NaCl) to individually stabilized Au NPs after PEG-grafting.

Synthesis of plasmonic nanoparticle markers for detection of *C. sakazakii* following the procedure sketched in Figure 1 started with the synthesis of citrate-stabilized 40 nm gold NPs using a seed-mediated approach described by Park et al. [36]. This synthesis leads to monodisperse particles, because the total number of Au nanoparticles is equal to the number of seed particles that are formed in the initial stage. The production of seeds stops before the growth phase. During the growth phase reduction of HAuCl_4_ by citrate on the seeds grows the particle size [38]. The Au nanoparticle size and morphology is controlled by the sodium citrate/HAuCl_4_ ratio [39]. A representative transmission electron micrograph of the as-synthesized Au NPs shows their spherical shape in Figure 2A; the size distribution of Au NPs was determined by image analysis of ~100 Au NPs to be 36 ± 7 nm in diameter. The gold cores were grafted with a polymer brush of mPEG(5 kDa)-HS, yielding colloidally stable Au NPs as shown earlier [37]. Functionalization of citrate-capped Au nanoparticles is relatively easy due to the weak electrostatic Au-citrate interaction. Ligand replacement can therefore be accomplished simply by reacting the Au nanoparticle with a surface excess of thiol-PEG, taking advantage of the relatively high affinity of the sulfur bond to gold [40]. The reaction at the Au nanoparticle surface, between a thiol group and the Au nanoparticle results in Au substituting the hydrogen from the thiol group. The PEG-brush surface modification prevents nonspecific protein adsorption, which improves IgG functionalization and reduces nonspecific binding to bacteria. The polymer grafting density and chain length in relation to particle size are important parameters governing the suppression of protein adsorption [41,42,43]. A PEG molecular weight of 5 kDa was chosen as this has been shown to produce a sufficiently thick brush to screen out nonspecific protein adsorption to the particle core [37], while grafting of higher molecular weight PEG can lead to insufficient grafting densities. We used a mixture of 0 to 100% HS-PEG-biotin/mPEG-HS as passivating ligands to produce gold NPs with a controlled variable density of terminal biotin as end-group for further biofunctionalization. The biotin end-groups of the PEG were used to anchor the biotinylated anti-*C. sakazakii* to the gold NPs via the strong streptavidin-biotin linkage [44,45].

The hydrodynamic diameter of the Au NPs after each step of functionalization was measured by DLS (Figure 2B). As synthesized citrate-stabilized Au NPs had a hydrodynamic diameter of 36.4 ± 7 nm, which increased by ~20 nm after grafting of the PEG brush. A PEG brush of this molecular weight is expected to be ~10 nm thick; the increase in hydrodynamic size after grafting of the PEG is therefore in agreement with the grafting of individual PEG-brushes to colloidally stable Au nanoparticles. Increasing concentration of biotin-terminated PEG chains led to further increase in the average hydrodynamic diameter by a few nm (Figure 2B); this is likely due to weak aggregation through the slightly hydrophobic biotin moiety. Electrophoretic measurement of as-synthesized citrate (charge) stabilized Au NPs yielded an expected negative Zeta potential of −34.6 ± 0.6 mV. The negative surface potential helps to prevent the Au NPs from aggregating and precipitating over time by providing a repulsive double-layer interaction [46]. Thereby, it facilitated further functionalization by grafting of the PEG brush. The Zeta potential became close to neutral after functionalization with mPEG-HS at −7.6 ± 0.3 mV. Adding a fraction of HS-PEG-biotin led to stronger negatively charged Au NPs, but the negative Zeta potential was reduced as the HS-PEG-biotin fraction was increased, e.g., −25.2 ± 1.1 mV for Au NPs functionalized with 5% HS-PEG-Biotin and −14.2 ± 0.6 mV for Au NPs functionalized with 100% HS-PEG-Biotin (see Table S2). However, the stability of almost neutral Au NPs coated with PEG is assured by the steric repulsion of the PEG brush.

The colloidal stability of gold nanoparticles can also be sensitively investigated by extinction spectroscopy thanks to their size-dependent plasmon polariton optical resonance. Aggregated nanoparticles experience a strong shift in and broadening of the plasmon resonance. Also binding of molecules to the surface results in a shift of the plasmon resonance to longer wavelengths through an increase in the refractive index within the evanescent field of the plasmon polariton. Figure 2C shows the extinction spectra of as-synthesized, mPEG-grafted and biotin-PEG-grafted gold NPs in 145.4 mM NaCl chosen to correspond to the physiological saline solution (0.85% NaCl). The spectrum of unmodified gold nanoparticles shows a broadened extinction peak at 527 nm, roughly corresponding to the expected plasmon resonance of the spherical 40 nm in diameter gold cores. However, there is also a broad second extinction peak at longer wavelengths. The broadened low wavelength peak and the shifted long wavelength peak point toward significant particle-particle interaction and aggregation in the sample of non-stabilized cores. Grafting of a PEG brush to the gold core does not seem to greatly change the peak value of the observed single-particle resonance (Figure 2C), but after grafting of PEG there is no sign of particle aggregation, i.e., the single-particle resonance has sharpened and the peak at long wavelengths is no longer present. The steric repulsion of the PEG brush is required to fully stabilize the nanoparticles at physiological ionic strength, and is thus a pre-requisite for biotechnological applications. Direct conjugation of antibodies to the nanoparticle surface will risk significant nonspecific van der Waals and charge interactions. Evidently, the PEG brush is strongly hydrated, since hardly any red-shift and broadening of the resonance peak is observed. Grafting of PEG end-functionalized with biotin does not significantly change the plasmon extinction spectrum of the gold nanoparticles; both 5% and 100% biotin-PEG show close to identical extinction spectra to 100% mPEG-functionalized NPs. The effect of steric stabilization *vs* surface charge stabilization was further investigated by changing the ionic strength in water from 0 to 145.4 mM NaCl (Figure S5). This shows that the Au nanoparticles grafted with a dense PEG brush are completely sterically stabilized and not dependent on the residual negative zeta potential of the biotin-PEG functionalized Au nanoparticles measured at low ionic strength, while the citrate-stabilized nanoparticles rapidly aggregate as the ionic strength is increased.

**Figure 3:**
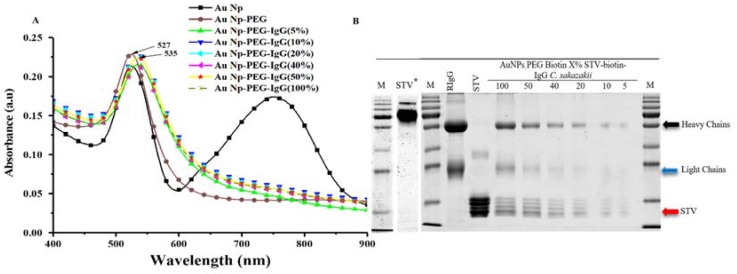
Characterization of optical properties and protein-functionalization of Au nanoparticles grafted with HS-PEG-biotin. (**A**) UV-Vis extinction spectra of Au-core NPs grafted with PEG brushes and functionalized with different percentages of biotin end-groups binding to streptavidin and further binding biotinylated anti-*C. sakazakii* IgG suspended in 145.4 mM of NaCl; (**B**) detection of anti-*C. sakazakii* IgG bound to Au nanoparticles functionalized with different percentages of biotin end-groups and streptavidin by SDS-PAGE. Two controls of streptavidin: one set was mixed with non-reducing sample buffer (non-denaturing) and the other was mixed with reducing agent (denaturing). M: marker, STV*: non-reduced streptavidin, RIgG: standard Rabbit Immunoglobulin G from rabbit serum (commercial), STV: reduced streptavidin, black and blue arrows indicate the band of heavy and light chains of the antibody respectively; the red arrow indicates the band of streptavidin.

The PEG brush also serves to prevent adsorption of the functional ligand, in this case the anti-*C. sakazakii*, directly to the surface of the nanoparticle, which in addition to nanoparticle aggregation can lead to denaturation and loss of function. Coupling of the functional group through a flexible linker like the PEG is also advantageous to enhance the steric accessibility of any ligand to the target. In this case, we improve the accessibility of anti-*C. sakazakii* IgG to target bacteria by linking it biochemically via a streptavidin spacer to the PEG-biotin-grafted Au NP core. For this, we first chemically modified the IgG with biotin groups. The functionalization of the NPs by the streptavidin-linked anti-*C. sakazakii* IgG could also be determined by UV-Vis spectroscopy. The extinction spectra comparing PEG-grafted Au nanoparticles with different percentages of biotin end-groups coupling the streptavidin-IgG complex are shown in Figure 3A. The prominent surface plasmon resonance extinction peak red-shifts from 527 nm to 535 nm as the streptavidin-IgG is coupled. We observed that already at 5% functionalization the spectral shift of the plasmon resonance peak was at maximum and did not increase at a higher percentage of functionalization. The red shift can be attributed to the increased effective refractive index within the localized evanescent field of the Au nanoparticle plasmon polariton, as a large number of proteins with higher refractive index than water are bound to the polymer brush. Extinction spectroscopy has previously been used as a method to confirm the successful conjugation of protein to gold NPs [47,48]. For example, Wang and Ni detected a shift in the plasmon resonance peak of 2–4 nm as the result of conjugation of protein to the gold nanoparticle surface [49]. The large wavelength shift of the peak maximum already at the lowest percentage of HS-PEG-biotin, which is not increased at higher degrees of functionalization, indicates that full coverage of a dense layer of anti-*C. sakazakii* is already reached. It is also worth noting that there is no indication of a second extinction peak at long wavelengths corresponding to nanoparticle aggregation after antibody coupling.

That the change in refractive index of the polymer brush grafted to the Au nanoparticle was due to the coupling of IgG was confirmed by treating the purified functionalized NPs after removal of excess IgG in solution by SDS to denature bound protein and to separate them by SDS-PAGE. As seen in Figure 3B, the bands corresponding to streptavidin and IgG are clearly identifiable for functionalized particles. The strength of the bands, corresponding to the relative amounts of protein found in the respective sample, correlate strongly with the biotin end-group functionalization of the nanoparticles; the higher the density of streptavidin on the surface of the particles, the higher the amount of protein (streptavidin and IgG) coupled to the particles. Thus, clearly, only biotin-streptavidin linked anti-*C. sakazakii* IgG is present on the particles after purification and the biotin end-group fraction in the PEG brush can be used to tune the ligand density. The spectroscopy measurement of the IgG functionalization did not reveal this strong dependence on HS-PEG-biotin fraction, which could be due to the fact that the PEG brush fills up most of the sensing volume of the highly localized plasmon polariton of the Au NP core. The plasmon resonance is only sensitive to the change in refractive index within quite a short distance from the particle surface. It is highly plausible that only part of the streptavidin linker layer and not the IgG layer influences the observed shift in plasmon resonance wavelength and that this proximal layer is less changed by the increasing amounts of coupled protein demonstrated by SDS-PAGE. However, in summary, all our data supports the conclusion that the Au NPs were successfully functionalized with anti-*C. sakazakii* IgG with retained colloidal stability under physiological conditions.

**Figure 4:**
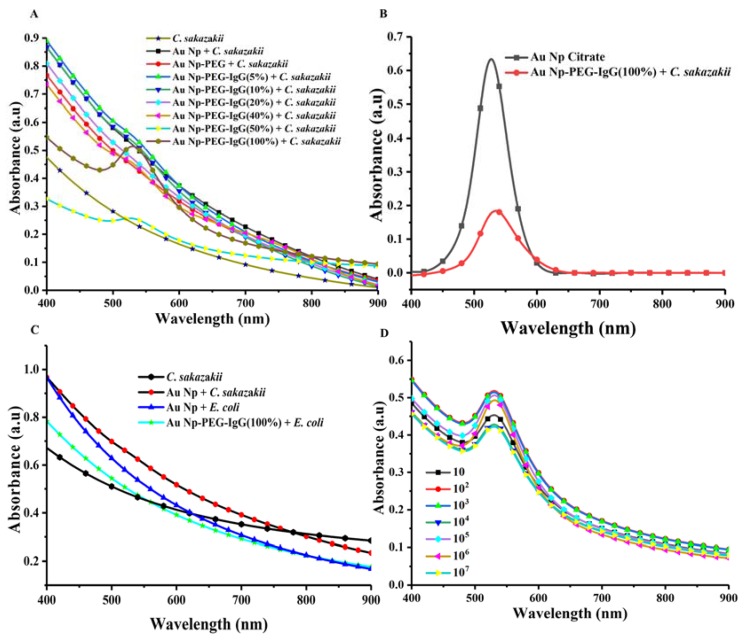
UV-Vis extinction spectra of *C. sakazakii* incubated with nanoparticles. (**A**) *C. sakazakii* at a concentration of 10^3^ CFU/mL incubated with targeted Au nanoparticles, functionalized with different percentages of biotin end-groups (HS-PEG-biotin 0–100%) and functionalized with biotinylated anti-*C. sakazakii* IgG via streptavidin. The samples were incubated for 2 h at room temperature and excess free particles thereafter were removed by filtration; (**B**) *C. sakazakii* at a concentration of 10^3^ CFU/mL incubated with targeted Au nanoparticles stabilized with 100% HS-PEG-biotin and functionalized with biotinylated anti-*C. sakazakii* IgG via streptavidin. The sample was incubated for 2 h at room temperature and excess free particles thereafter were removed by filtration. The background of a sample containing only *C. sakazakii* was subtracted to reveal the extinction peak of the targeted Au nanoparticles. Also, the extinction peak of a pure dispersion of functionalized Au nanoparticles at known initial concentration with baseline correction; (**C**) negative controls of *C. sakazakii* and *E. coli* incubated with PEGylated Au NPs followed by removal of the free particles by filtration as well as *C. sakazakii* without Au NPs. Additionally, *E. coli* incubated with Au NPs 100% functionalized with anti-*C. sakazakii*, following the same procedure and conditions as in (**A**). (**D**) *C. sakazakii* at different concentrations 10^1^ to 10^7^ CFU/mL incubated with Au nanoparticles stabilized with 100% HS-PEG-biotin 100% and functionalized with biotinylated anti-*C. sakazakii* IgG via streptavidin. Please note that the sample containing 10^7^ CFU/mL was diluted 25 times for the absorption measurement due to high background scattering.

After demonstrating successful functionalization, we tested the immunogold particles for targeting and spectroscopic detection of *C. sakazakii*. After incubation of the nanoparticles with *C. sakazakii* for 2 h at room temperature in a shaking incubator followed by separation of the bacteria from free nanoparticles by filtration, the suspension of bacteria was investigated by extinction spectroscopy. Figure 4A shows a comparison of extinction spectra for *C. sakazakii* incubated with IgG-functionalized nanoparticles with different PEG-biotin fractions after filtration to remove free nanoparticles from the dispersion. Comparing these spectra to that of pure *C. sakazakii* reveals a peak or shoulder roughly corresponding to the position where the plasmon resonance of the Au NPs appeared for the pure NP dispersions. To further verify and visualize the nanoparticle peak of the labeled bacteria samples, it is instructive to subtract the background absorption of the bacteria and the additional background recorded for the functionalized nanoparticles that is not related to the plasmon polariton extinction peak. Figure 4B shows the spectrum of the pure nanoparticles compared to the spectrum of the separated *C. sakazakii* bacteria incubated with targeted 100% PEG-biotin functionalized nanoparticles; the latter has the background spectrum of the corresponding pure *C. sakazakii* bacteria sample subtracted. A comparison shows that the peak appearing in the incubated and purified *C. sakazakii* sample corresponds to the plasmon extinction peak of the targeted nanoparticles. The nanoparticles bind to *C. sakazakii* and therefore the signal is specific, as evidenced by the fact that no corresponding plasmon peak was found for a control sample of the related food pathogen *E. coli* incubated with the anti-*C. sakazakii* functionalized nanoparticles. This demonstrates that the antibodies retain the specificity demonstrated by Western blot also after coupling to the Au NPs. Figure 4C shows the result of the *E. coli* negative control as well as the background spectra of the pure bacteria suspension and the control for incubating *C. sakazakii* with PEG-grafted nanoparticles that were not functionalized with anti-*C. sakazakii*. It is thus evident that we, by the PEG-grafting, managed to avoid nonspecific labeling of bacteria and thereby achieve specific labeling using the functionalization with anti-*C. sakazakii*.

Figure 4A also shows the extinction spectra as a function of degree of NP functionalization. A clear dependence on the fraction of IgG coupled to the nanoparticles is observed. The 100% PEG-biotin grafted Au NPs functionalized with IgG show a much more prominent plasmon extinction peak than the lower degrees of functionalization. The background can be subtracted from the plasmon peak in the extinction spectra, as demonstrated in Figure 4B, and thereby enables a quantitative comparison of the nanoparticle binding to bacteria for different degrees of functionalization. The area of the extinction peak corresponds to the total plasmonic scattering of the sample emanating only from Au nanoparticles. It should therefore be proportional to the concentration of the nanoparticles in the sample and can be compared between the samples. Importantly, it can be compared between the bacteria samples labeled with Au NP and a sample containing a known concentration of functionalized nanoparticles to quantify the number of Au nanoparticles in any sample. This analysis benefits from the fact that nanoparticles after functionalization with a stabilizing PEG brush differ very little in their scattering when the local environment changes, e.g., by binding to a bacterial surface; this is demonstrated by the absence of change in the extinction peak for nanoparticles with different degrees of streptavidin-IgG functionalization (cf. Figure 3A). We performed the quantitative analysis on the plasmon extinction peak data in Figure 4A treated as in Figure 4B to compare the concentration of nanoparticles remaining bound to the bacteria after the targeting and separation steps. The results are listed in Table 1 and show that a high fraction of the particles are bound to the bacteria after separation of unbound particles by filtration. The degree of functionalization influences the number of bound particles, with the 100% biotinylated particles (the highest degree of functionalization) yielding half of the particles in the assay strongly bound to the pathogen. The difference between the lowest and highest degree of functionalization is one order of magnitude. However, it is noteworthy that the fraction of particles that are irreversibly targeted to *C. sakazakii* is fairly constant, except for the highest degree of functionalization.

That not all targeted particles are found bound to bacteria in the separated sample and that a dependence on degree of functionalization is observed could be explained in two different ways that cannot be clearly distinguished based on the data. First, it is possible that when the degree of functionalization is low, fewer nanoparticles carry even one IgG that can bind to the bacteria, since the number of IgG per Au nanoparticle will be statistically distributed. This is unlikely as even nanoparticles with 5% PEG-biotin have plenty of binding sites, and the extinction spectroscopy and SDS-PAGE results indicate a high amount of protein bound to the nanoparticles. However, it is possible that biotinylation reduces the binding affinity of IgG, since the sites of biotinylation using our approach are not controlled and can also occur in the area of the binding sites. The binding to the nanoparticle surface can also result in non-active orientations that are sterically blocking the binding sites for a fraction of IgG. Thus, also nanoparticles with several bound IgG could have a very low effective avidity due to carrying IgG that are sterically blocked or have reduced affinity. Second, it is possible that the antibodies do not have sufficient affinity to bind over the time period of the purification step. Antibodies often do not have an affinity high enough for the longer-than-minute time-scale binding that is required for the separation step; multivalent binding of anti-*C. sakazakii* coupled to nanoparticles to the surface of the bacteria would then be required for detection after the separation and washing steps. This is a strong possibility if the antibodies target, e.g., polysaccharide motives on the surface of the bacteria. The polyclonal antibodies could also target different epitopes on the bacteria surface with different affinities. As a result, a higher degree of functionalization promotes binding of more nanoparticles that can be detected after separation of the sample from free nanoparticles. This is the more likely explanation of our observation. In summary, our results indicate that only a fraction of the functionalized nanoparticles possess an affinity sufficient for irreversible binding and that this fraction increases when the density of IgG on the particle surface is increased. Also, for the nanoparticles with the highest degree of functionalization, only a fraction are irreversibly bound. However, these particles accumulate on the bacteria during incubation and the sensitive plasmonically-enhanced readout scheme can detect also a low number of nanoparticles remaining bound to bacteria in the sample after separation of weakly bound particles.

Finally, using the most efficient particles for targeting, with maximum functionalization, we tested the targeting of samples containing different concentrations of *C. sakazakii* obtained by serial dilution. The extinction curves for bacteria suspensions containing ~1 × 10^1^ to 1 × 10^7^ CFU/mL incubated with 100% HS-PEG-biotin and functionalized with biotinylated anti-*C. sakazakii* IgG via streptavidin are shown in Figure 4D. As can be seen from this data, a high plasmon extinction signal is recorded also at the lowest tested concentrations of *C. sakazakii*. Indeed, the signal is almost independent of pathogen concentration, easily achieving a detection limit as low as 10 CFU/mL with the fully functionalized nanoparticles. Each bacterial cell has a large surface area relative the nanoparticles and can presumably be targeted by a large number of nanoparticles. We are therefore observing that an increased concentration of irreversibly binding nanoparticles leads to more nanoparticles binding per bacterium and therefore to a higher signal per bacterium, while increasing the concentration of bacteria at the used nanoparticle concentration changes the concentration of nanoparticles detected bound to bacteria surfaces by a much smaller amount (cf. Figure S7). This indicates that for specifically and irreversibly binding particles with multiple possible binding sites, like those designed for this study, the signal and ease of detection of few bacteria are increased compared to bacteria in samples with high concentrations of the target organism. In other words, our proof of concept is suitable for detection of few bacteria, which is important for applications in the food industry where verification of complete absence of *C. sakazakii* in food samples is required. We can, in this way, only demonstrate sensitive specific binding. The selectivity of the assay for *C. sakazakii* in a complex environment containing also other species can only be assumed based on the fact that no binding to other bacteria was observed of the IgG-functionalized nanoparticles nor of the polyclonal antibodies in separate tests on pure cultures.

For practical reasons, we could not perform measurements to test if single bacteria sensitivity can be reached in smaller analyzed volumes. This is not possible with the setup used in this study, which on purpose was a non-optimized approach using standard lab equipment available in any lab. Our proof of concept has the advantage of presumably leading to a low-cost and rapid assay since low-cost spectrometers for a few hundred Euros provide sufficient sensitivity and the tests can be rapidly performed on site by lesser-trained personnel. For example, an LFA strip-based readout, as mentioned in the introduction, can be even lower cost and easier to use, but at the expense of a higher limit of detection [18]. PCR analysis requires lab equipment that are orders of magnitude more expensive plus they require more time of skilled personnel. An optimized and miniaturized spectroscopic readout is likely to increase the sensitivity of our assay down to single bacteria and could lead to even more cost-effective setups similar in use to the strip-based approach, with the difference that they would also be fully reusable. Single plasmonic particle readout schemes in confined volumes have been reported in the literature and we achieve tagging of bacteria with multiple nanoparticles through the efficient mixing of probe and sample combined with high-affinity nanoparticles. This should also enable single bacterium detection. A flow-cytometric approach could be used to study the response per bacterium, as well as to sort bacteria and to verify the performance on *C. sakazakii* in mixed cultures. With such additional information, it would also be possible to calibrate the assay for quantitative detection over a larger concentration range in addition to achieving highly sensitive detection by using nanoparticle-based extinction spectroscopy. This requires confirming individual tagging of bacteria by TEM in combination with the spectroscopic detection. This will be the subject of further study also aiming at single pathogen detection.

If required, further increase in signal-to-noise could also be achieved by testing larger Au nanoparticles that scatter light at longer wavelengths where there is less background absorption from the biological sample. However, larger nanoparticles are more difficult to keep colloidally stable in complex media and more difficult to separate. These potential drawbacks would have to be traded against the lower background that can facilitate detection. Although this estimate is based on the bacteria dilution series without background from other bacteria, the control using an *E. coli* sample demonstrated no discernable signal from nanoparticles binding to the *E. coli*. This indicates that nonspecific background signal from binding to other bacteria can be kept very low or, as shown here, negligible, using proper functionalization of the nanoparticle surface.

In terms of sensitivity, ease and speed of use, the performance of the presented detection scheme can be compared to the previous examples of rapid and sensitive detection of *C. sakazakii* without the use of specialized equipment that we described in the introduction. The LFA strip using a sandwich assay of Au nanoparticles with monoclonal antibodies specific to *C. sakazakii*, achieved assay times after preparation of the strips and nanoparticles significantly shorter than 1 h using a very simple readout format that can be used anywhere. However, the detection limit was probably bounded by the background signal from nonspecific binding and especially by the less efficient targeting obtained from a macroscopic surface compared to targeting in the bulk using nanoparticles; hence the limit of detection was two orders of magnitude higher than in our assay at >10^3^ CFU/mL [18]. A similar detection limit of ~10^3^–10^4^ CFU/mL was achieved using magnetic immunoliposomes and a fluorescence-based readout [8]. The detection limit in this case seemed to be compromised by high background signal, which could be due to the fact that the fluorescence reporter is associated with liposomes containing both the dye and magnetic nanoparticles. This construct has much lower stability than PEG-grafted nanoparticles. The clever and simple magnetic separation method, however, led to an assay time similar to ours, i.e., ~2.5 h. Hence, we achieved an orders of magnitude lower limit of detection using a comparable assay time and ubiquitous equipment that could be used for on-site specific detection of *C. sakazakii* without special training. However, in its current implementation this performance comes at the expense of an absence of a linear detection range for direct quantification of pathogen concentration.

**Figure 5:**
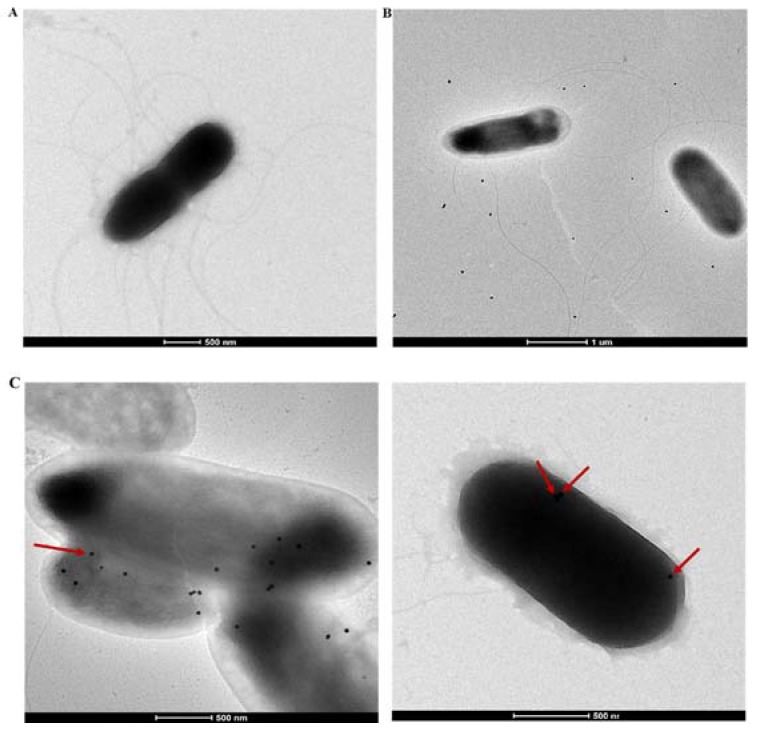
Transmission electron micrographs of *C. sakazakii*. (**A**) *C. sakazakii*; (**B**) *C. sakazakii* incubated with PEG-grafted Au nanoparticles. The nanoparticles are only observed in the background; (**C**) two micrographs of *C. sakazakii* incubated with targeted Au nanoparticles stabilized with 100% HS-PEG-biotin and functionalized with biotinylated anti-*C. sakazakii* IgG via streptavidin. The samples were incubated for 2 h at room temperature and excess free particles thereafter were removed by filtration. The nanoparticles are found bound to the bacteria (identified by red arrows) in a manner consistent with binding to the cell membrane.

Finally, to verify that the nanoparticles are bound to the surface of the bacteria and are not part of the background or internalized, we also performed transmission electron microscopy on bacteria from the incubated sample. Representative single isolates of *C. sakazakii* that have the expected rod shape are shown in Figure 5A, but no features can be identified in the micrograph that are similar to the high electron density contrast and regular shapes of Au NPs. Addition of non-targeted but PEG-grafted Au nanoparticles resulted in no Au nanoparticles being detected by TEM (or extinction spectroscopy) after separation of free particles, but in a non-filtered sample we can image Au nanoparticles in the background but not bound to the bacterial cells (Figure 5B). Addition of the PEG-grafted Au NPs functionalized with anti-*C. sakazakii* IgG to a suspension containing *C. sakazakii*, resulted in the binding of the conjugate to the surface of the cell that could be imaged by TEM due to the high electron density of the gold nanoparticles compared to the organic background. The micrograph in Figure 5C shows easily identifiable gold nanoparticles, providing high electron density contrast distributed over the bacteria surface but not in the background; a comparison with Figure 5B demonstrates that targeting took place. The gold NPs are distributed uniformly over the *C. sakazakii* cell surface, as can be expected for colloidally stable and specifically targeted nanoparticles. Studying a large number of micrographs of NP-targeted bacteria samples we found a distribution of 2–9 gold nanoparticles attached per cell.

## 4. Conclusions

In summary, we have shown that gold nanoparticles functionalized with antibodies raised against *C. sakazakii* in rabbits can be used for highly sensitive and rapid specific detection of *C. sakazakii* with a UV-Vis spectrometric readout. Without optimization, using a simple spectrometer and cuvette setup, we could as proof of concept detect on the order of 10 *C. sakazakii* CFU/mL with a turnaround time of less than two hours. Importantly, stabilization of the nanoparticles with a dense PEG brush before IgG functionalization is required for the highly specific and reproducible targeting that enabled the assay, in comparison to previous results in the literature. It was shown that a higher density of functional groups on the nanoparticles resulted in a higher amount of coupled antibody. Furthermore, the easily discernible and strong extinction peak of monodisperse Au nanoparticles allows for a quantitative measure of the number of targeted nanoparticles in a sample that has undergone the separation and analysis steps. This analysis showed that the irreversibly and specifically binding nanoparticles led to an increased number of nanoparticles targeted per bacterium at decreased pathogen concentration, which resulted in a very low limit of detection. However, it also compromised the ability to quantify the concentration of bacteria in a dispersion based on the extinction spectra alone, since the average number of nanoparticles per bacterium varied strongly. Although the assay is already rapid and sensitive using standard lab equipment, it could easily be optimized in the future in terms of higher throughput and sensitivity, which is likely to reach single pathogen detection using miniaturized sample handling and readout schemes.

## Figures and Tables

**Table 1:** Calculation of concentrations of targeted nanoparticles in *C. sakazakii* samples using the plasmonic extinction peak and the corresponding fractions of particles bound to bacteria for the data in Figure 4A.

Sample	Area (a.u.) *	Concentration (pM) ***	Percentage Particles Bound (%)
AuNP-PEG-IgG (100%)	43.5 **	1.2	100
AuNP-PEG-IgG (5%) + *C. sakazakii*	2.08	5.7 × 10^−2^	4.8
AuNP-PEG-IgG (10%) + *C. sakazakii*	3.65	0.10	8.4
AuNP-PEG-IgG (20%) + *C. sakazakii*	0.443	1.2 × 10^−2^	1.0
AuNP-PEG-IgG (40%) + *C. sakazakii*	5.49	0.15	13
AuNP-PEG-IgG (50%) + *C. sakazakii*	4.28	0.12	9.8
AuNP-PEG-IgG (100%) + *C. sakazakii*	21.9	0.60	50

* Area of the extinction peak of Au nanoparticles after subtraction of *C. sakazakii* and cell background; ** Area of extinction peak of pure nanoparticle solution without *C. sakazakii* background subtraction; *** Concentration of gold targeting bacteria = AuNP Citrate concentration × Area of Au nanoparticle extinction peaks functionalized after background subtraction/Area of AuNP Citrate. Please note that the concentration of AuNP used to normalize these concentrations is an estimate based on an assumed conversion of all precursor into Au NP during synthesis and purification.

## Supplementary Materials

The following are available online at http://www.mdpi.com/1424-8220/18/7/2028/s1, Detailed protocols and additional data and results. Figure S1: SDS-PAGE of purified anti-*C. sakazakii* IgG, Figure S2: Western blot of whole bacterial cell material of *C. sakazakii*, Figure S3: DLS size distributions fo differentl functionalized Au nanoparticles, Figure S4: TGA curve of weight loss upon heating the Au nanoparticles functionalized with HS-PEG-biotin 100%, Figure S5: UV-Vis extinction spectroscopy of differently functionalized Au nanoparticles, Figure S6: UV-Vis extinction spectra of bacteria samples of *C. sakazakii* incubated with PEG-grafted Au nanoparticles and then separated from free Au nanoparticles, Figure S7: Bacteria targeting by Au nanoparticles, Table S1: Zeta Potential determination of gold nanoparticles, Table S2: Zeta potential determination of gold nanoparticles.
