# Supplementary material for: Immunogold Nanoparticles for Rapid Plasmonic Detection of C. sakazakii
Source: Sensors (Basel). 2018 Jun 25;18(7):2028. doi: 10.3390/s18072028 (PMC6068645; doi:10.3390/s18072028)
Supplement: Supplementary file 1 [file sensors-18-02028-s001.pdf]

sensors

Supplementary Materials: Immunogold Nanoparticles for Rapid Plasmonic Detection of *C. sakazakii*

Mohamed A. Aly, Konrad J. Domig, Wolfgang Kneifel and Erik Reimhult

Content

1. Antibody Production

1.1. Immunogen and standard preparation

1.2. Immunization

1.3. Purity of prepared rabbit anti-*C. sakazakii* antibody

1.4. Western blot

2. Particle synthesis and characterization

2.1. Gold NPs synthesis

2.2. Gold NP size and concentration determination

2.3. Thermogravimetric analysis (TGA) of PEG grafting density

1. Antibody Production

1.1. Immunogen and standard preparation

Cronobacter sakazakii (DSM-4485) was chosen as an immunogen for the preparation of rabbit antibodies. The microorganism was cultivated in 500 mL of TSB at 37 °C for 24 h. After cultivation, the cells were harvested by centrifugation at 8000 g for 10 min at 4 °C. Then, pellets were washed three times with saline 0.85% NaCl solution and resuspended to the final volume of 25 mL. The resuspended cells were boiled for 1 h followed by centrifugation and resuspension in 25 mL of saline and stored at 4 °C for 24 hours.

1.2.Immunization

New Zealand White rabbits were challenged with *C. sakazakii*. Before each immunization antigens were thawed and 4 mL of antigen were collected in a tube; 700 µL of complete Freund's Adjuvant (FA) were added. Complete FA was added for the first immunization and incomplete FA for the 2nd and 3rd immunizations. The repeat immunizations took place at 3-week intervals. Blood was collected from the ear vein (small blood amounts) or from the heart (terminal bleeding). Blood samples were incubated at 4 °C for 1–2 hours with subsequent centrifugation 10.000 g twice for 5 minutes. Individual serum samples were stored at -20 °C.

1.3. Purity of prepared rabbit anti-C. sakazakii antibody

The purity of the anti-*C. sakazakii* antibody was checked using SDS-PAGE under reduced conditions as described by [1] with minor modifications. A polyacrylamide stacking gel (4% w/v) and a separating gel (15% w/v) were used to separate the distinct proteins into bands. Briefly, the samples were mixed with equal volume of digestion buffer yielding the final concentrations: 0.125 M Tris-

HCl, 2% (w/v) SDS, 5% (v/v) glycerol, 5% (v/v) 2-mercaptoethanol, and 0.002% (w/v) bromophenol blue. Samples were boiled for 3 min at 100 °C for complete dissociation of proteins. Further, sample aliquots (8 μL) were loaded into each well. Pre-stained SDS-PAGE markers and standard rabbit antibody (IgG) were used as molecular weight markers and control, respectively. Electrophoresis was done at a constant voltage of 200 V and at 45 mA at room temperature. After SDS-PAGE, the gel was stained with Coomassie blue for at least 3 h. Visualization was carried out in Coomassie blue staining solution at room temperature. The destaining procedure was performed in solution of 40% methanol and 10% glacial acetic acid; the destaining solution was replaced three times until the background was nearly clear. After destaining, the gel was wash in deionized water (Figure S1).

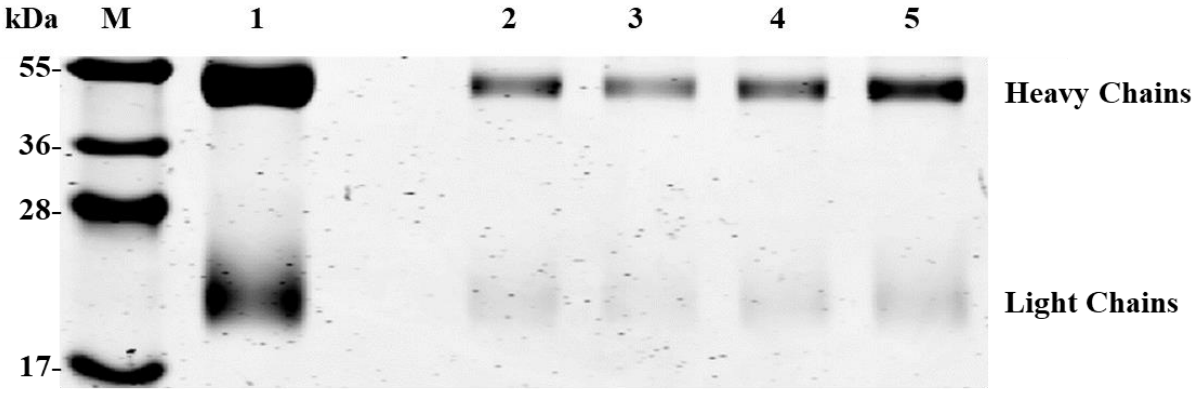

Figure S1. SDS-PAGE of purified anti-*Cronobacter sakazakii* IgG. Two bands at 50 kDa and 25 kDa were observed in lane 1 for the commercial rabbit IgG and lanes 2–5 for anti-*C. sakazakii* IgG, respectively. The molecular mass markers are indicated on the left.

1.4. Western blot

1 mL was kept in overnight culture at 37 °C. Then the cells were spun down and the pellet in 1x Laemmli 2% (w/v) SDS, 1% β-mercaptoethanol, 10% glycerol in 0.25 mM-Tris / HCl, pH 6.8 to a final concentration of 0.1 g/mL. The resuspended sample was boiled for 6 min and then spun at 8000 g for 10 min. 8 μL of the sample supernatant was loaded on the polyacrylamide gel SDS-PAGE. Electrophoresis followed at 170 V for 65 min. The separated proteins were transferred to the membrane polyvinylidene fluoride (PVDF) and soaked in methanol for activation. After transfer, the membrane was blocked with 5 % (w/v) skim milk in 1x PBS buffer for 3 h at room temperature and incubated with rabbit anti-*C. sakazakii* IgG 1:5000 solution containing 0.5% (w/v) skim milk in PBS buffer; incubation was done overnight at 4 °C. The membrane was washed five times in PBS containing 0.02% (v/v) Tween 20 and then reacted with IRDye 680RD Goat anti-Rabbit secondary antibody with 1:25000-dilution for conjugate antibodies in 1× PBS buffer containing 0.5% (w/v) skim milk. Subsequently, the membrane was washed 5 times in PBS containing 0.02% (v/v) Tween 20. Finally, the membrane was dried and then visualized (Figure S2).

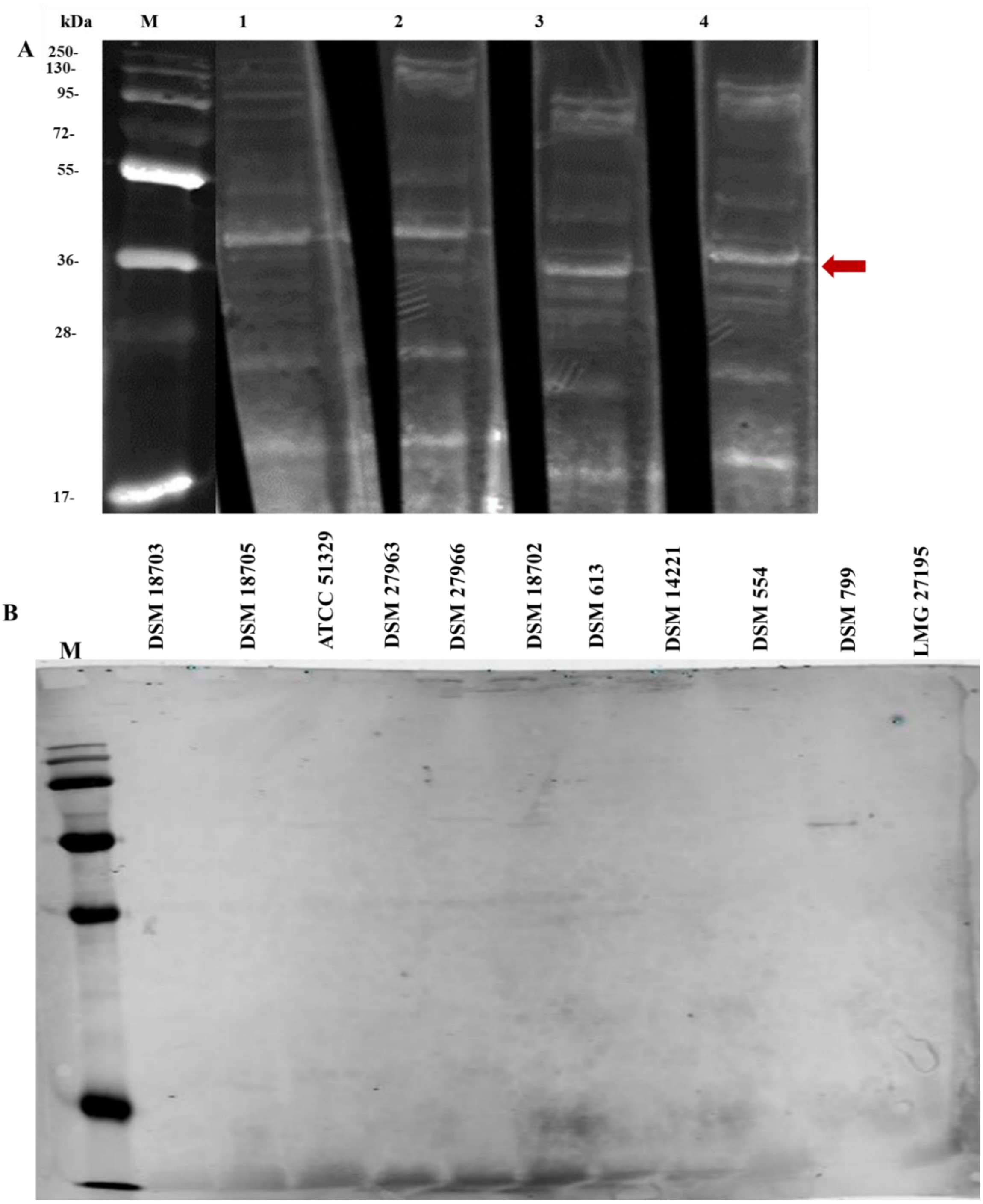

Figure S2. Western blot of whole bacterial cell material (A) of *C. sakazakii* probed for four different batches of rabbit IgG (anti-*C. sakazakii*). The arrowhead indicates the binding protein. (B) Western blot loading control of pathogenic bacteria. M is a marker and the red arrow indicates the band of antibody bound to bacteria after separation.

Table S1. Western blot analysis of the specificity of anti-*C. sakazakii*

Strain	Detection
Cronobacter sakazakii D	+
Cronobacter turicensis DSM 18703	-
Cronobacter dublinensis dublinensis DSM 18705	-
Cronobacter muytjensii ATCC 51329	-
Cronobacter universalis DSM 27963	-
Cronobacter condimenti DSM 27966	-
Cronobacter malonaticus DSM 18702	-
Salmonella Typhimurium DSM 554	-
Salmonella enterica subsp. *enterica* DSM 14221	-
Enterobacter xiangfangensis LMG 27195	-
Staphylococcus aureus subsp. *aureus* DSM 799	-
Escherichia coli DSM 613	-

2. Particle synthesis and characterization

2.1. Gold nanoparticle (NP) synthesis

Gold nanoparticles with a diameter of 40 nm were prepared by seed-mediated synthesis [2]. NP seeds were prepared by conventional citrate reduction of chloroauric acid: A 200 mL solution of $HAuCl_4$ 0.5 mM in water was heated to boiling; after that, 10 mL solution of sodium citrate 38.8 mM in water was added quickly. This mixture was then continuously boiled until the solution after approximately 20 minutes had developed a ruby red color. Seed-mediated growth was then performed at room temperature with ascorbic acid as reducing agent. An aqueous solution of 4 mL $HAuCl_4$ with concentration 20 mM, another aqueous solution of 0.4 mL $AgNO_3$ with concentration 10 mM and finally 15 mL of the pre-prepared seed NPs were all added to 170 mL Milli-Q water. An aqueous solution of in total 30 mL of ascorbic acid with concentration 5.3 mM was then added at a rate of 600 μL/min to the seed-solution under constant stirring.

2.2. Gold NP size and concentration determination

Citrate-stabilized gold NPs were functionalized with poly(ethylene glycol) (PEG) via self-assembly of thiol-PEG (MW 5 kDa) of which a part had a methyl terminal moiety and a part had a biotin terminal moiety. Prior to surface modification, the 40 nm NPs were purified three times by centrifugation and concentrated by centrifugation at 800 g for 90 min and then diluted to 1 nM with Milli-Q water. The modification was done by adding a suitable amount of an aqueous solution of thiol-PEG directly to test tubes with gold NP solution followed by thorough mixing and then incubation overnight at 4 °C under gentle shaking. To ensure saturated surface coverage of PEG; modification was done in the presence of an excess of thiolated PEG in relation to what is required for full surface coverage. The final concentration of thiol-PEG after mixing with NPs was adjusted to 10 thiolated PEG molecules per nm^2 available surface area in the NP solution [3], while the expected final grafting density is < 1 HS-PEG molecule per nm^2. The PEG-grafted gold NPs were kept as a highly concentrated stock solution. The NP concentration was determined by measuring the light

absorption at λ = 527 nm. Finally, the nanoparticles were diluted in NaCl (10 mM, pH 7.2) solution. Samples were incubated at room temperature for 5 min for DLS measurements of the hydrodynamic size of the as-synthesized and PEG-grafted NPs were made (Figure S3) and complemented by Zeta potential measurements (Table S1). All gold NPs were stored in the refrigerator at 4 °C until use.

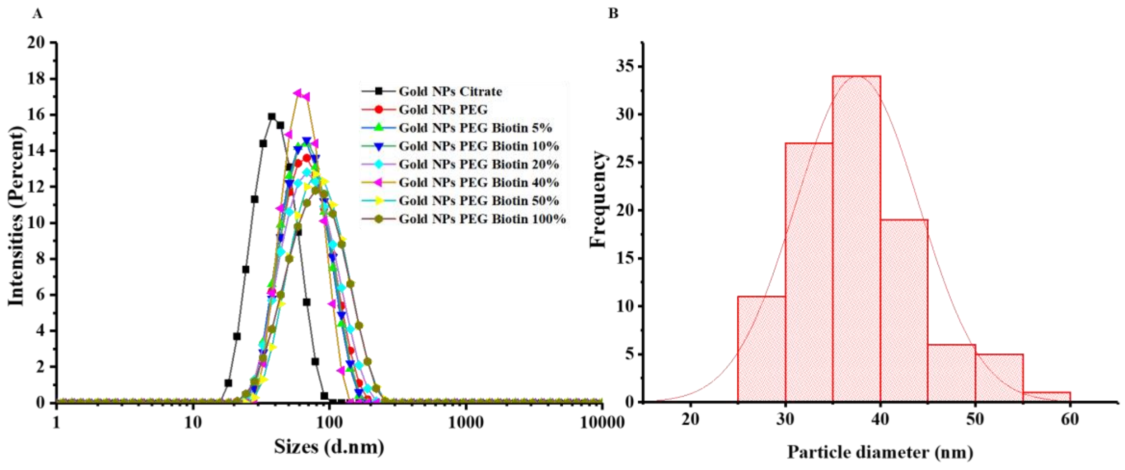

Figure S3. (**A**) Comparison of the intensity-weighted size distributions obtained by dynamic light scattering for gold nanoparticles with different surface ligand compostion. (**B**) Size distribuition of gold NPs citrate determined by ImageJ image analysis from TEM micrographs.

Table S2. Zeta potential determination of gold nanoparticles.

Sample	Zeta Potential (mV)
AuNPs citrate	-34.6 ± 0.6
AuNPs PEG	-7.6± 0.3
AuNPs PEG biotin 5%	-25.2 ± 1.1
AuNPs PEG biotin 100%	-14.2 ± 0.6

2.3. Thermogravimetric analysis (TGA) of PEG grafting density

To measure the grafting density of PEG on the NPs, thermogravimetric analysis (TGA) was performed. The gold NPs were extensively purified before freeze-drying to prepare the sample for TGA. 2 mg of 40 nm gold NPs was weighed into 70 μL AlOx-cups and analyzed with a Mettler-Toledo TGA/DSC 1 instrument. The sample was measured under constant flow of synthetic air (80 mL/min plus 20 mL nitrogen stream as protection gas for the balance) with a heating rate of 10 K/min and the ligand decomposition was estimated in the range 100 to 650 °C (Figure S4). For biotin-PEG-grafted 36-nm Au nanoparticles, a relative mass loss of approximately 6.24% was detected, which corresponds to an absolute mass of 7.5×10^{-17} g or 4153 HS-PEG-biotin molecules per NP. Using the average area per nanoparticle this results in a grafting density of 0.94 nm^{-2}.

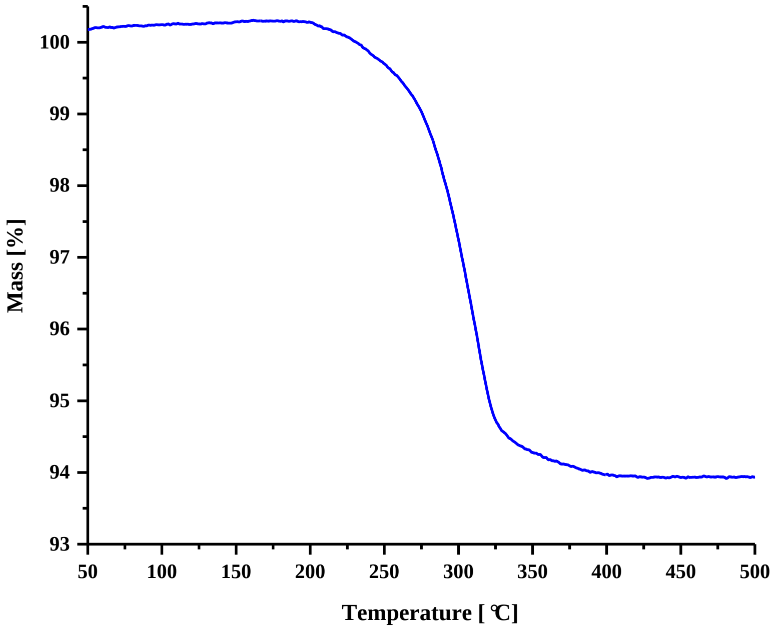

Figure S4. TGA curve of weight loss upon heating the Au nanoparticles functionalized with HS-PEG-biotin 100%.

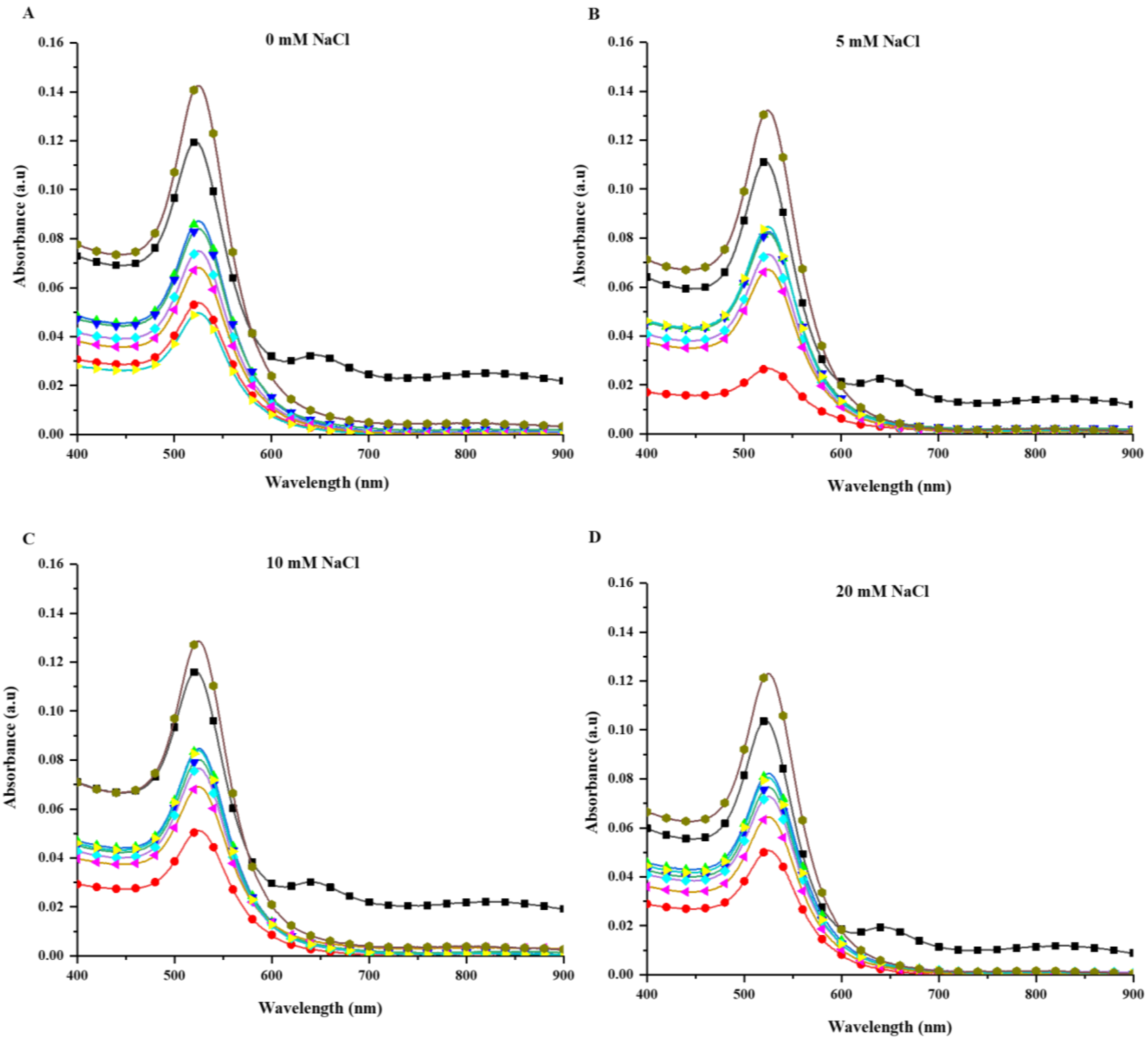
A
0 mM NaCl
B
5 mM NaCl
C
10 mM NaCl
D
20 mM NaCl
Absorbance (a.u)
Wavelength (nm)
0.00
0.02
0.04
0.06
0.08
0.10
0.12
0.14
0.16
400
500
600
700
800
900

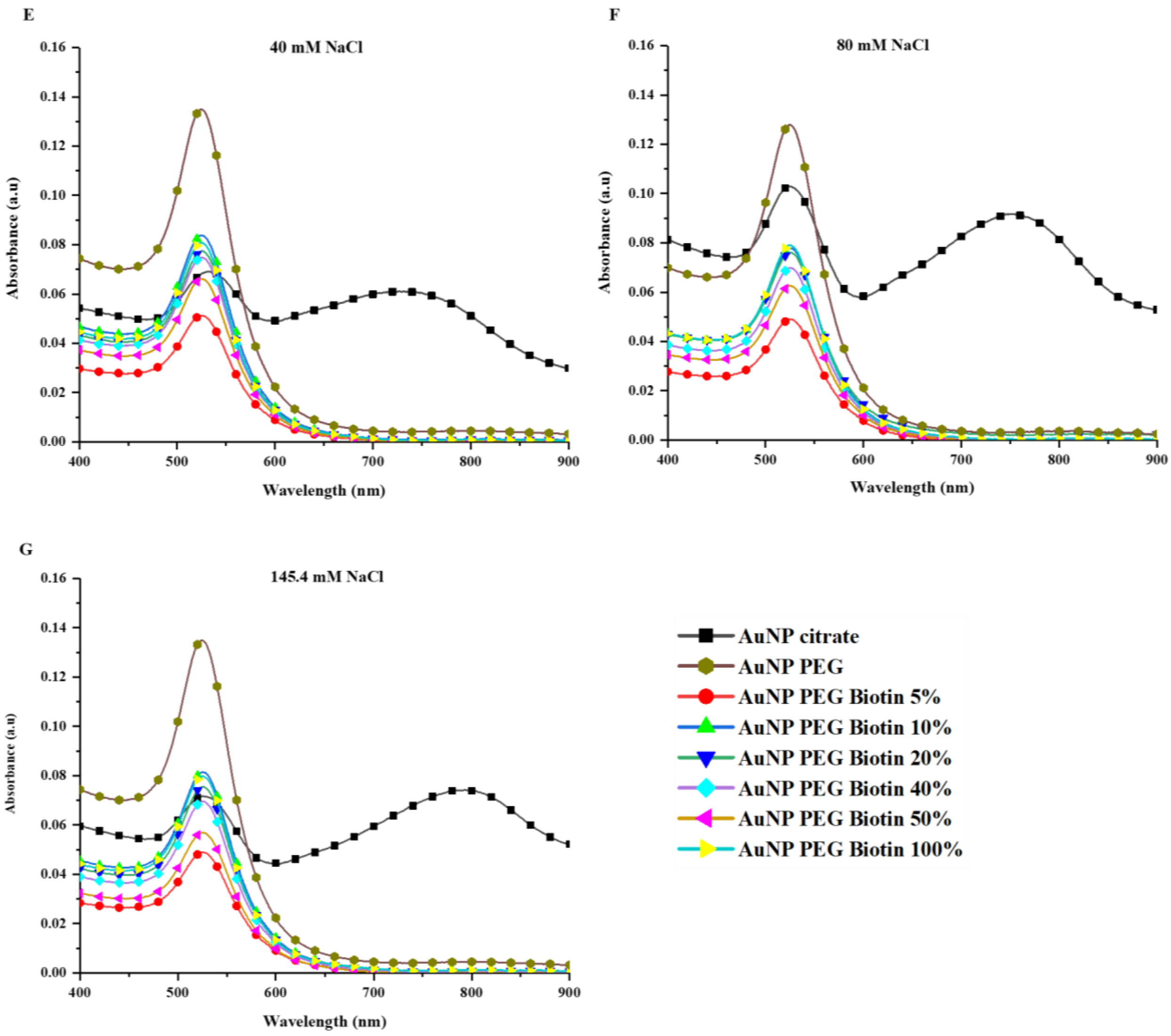

Figure S5. UV-Vis extinction spectroscopy of differently functionalized Au nanoparticles. The transmission spectra are shown for as-synthesized citrate stabilized Au nanoparticles and PEG-grafted nanoparticles for which the percentage of biotin-functionalized PEG was varied. (**A**)–(**G**) show these nanoparticles suspended at different ionic strength from 0 to 145.4 mM NaCl.

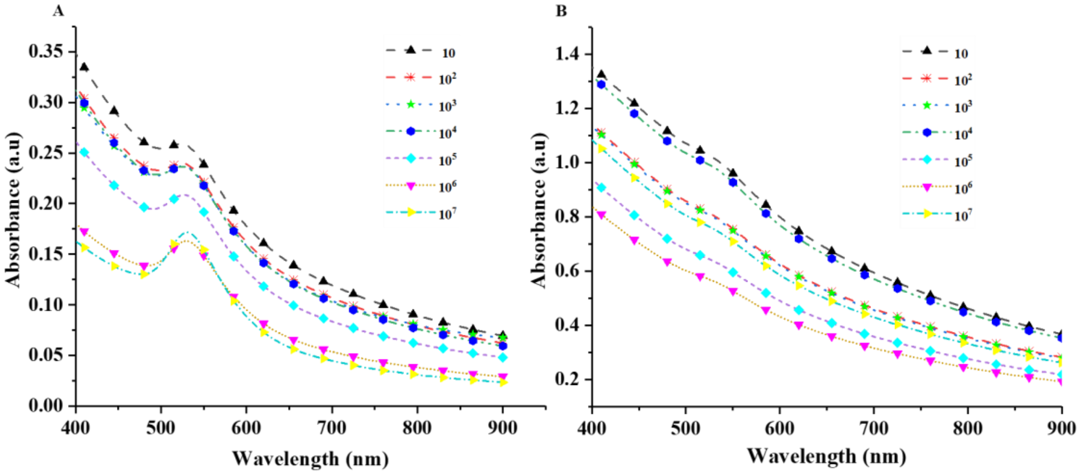

Figure S6. UV-Vis extinction spectra of bacteria samples of *C. sakazakii* incubated with PEG-grafted Au nanoparticles and then separated from free Au nanoparticles. The bacteria and nanoparticles were suspended in physiological saline buffer and incubated for 2 h at room temperature, and thereafter separated by filtration and resuspended for measurement by UV-Vis spectroscopy. (A) Extinction spectra of *C. sakazakii* at concentrations varied between 10^1–10^7 CFU/mL incubated with gold NPs functionalized with HS-PEG-Biotin 50% conjugated with the biotinylated anti-*C. sakazakii* IgG via streptavidin and (B) extinction spectra of *C. sakazakii* at concentrations varied between 10^1–10^7 CFU/mL incubated by gold NPs functionalized with HS-PEG-Biotin 40% without anti-*C. sakazakii* IgG.

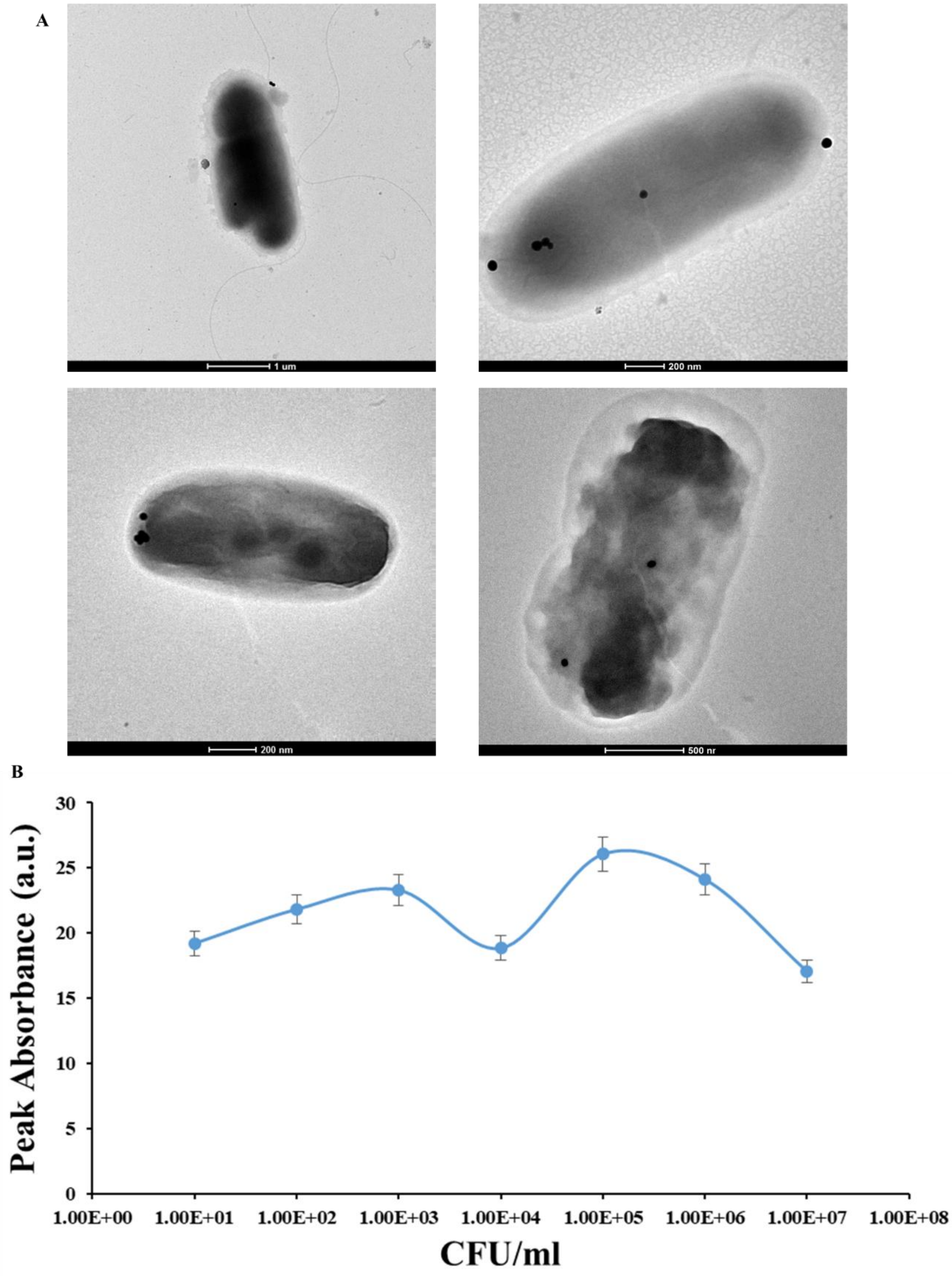

Figure S7. Bacteria targeting by Au nanoparticles. (**A**)Transmission electron micrographs of *C. sakazakii* incubated with targeted Au nanoparticles stabilized with 100% HS-PEG-biotin and functionalized with biotinylated anti-*C. sakazakii* IgG via streptavidin. (**B**) Integrated peak absorbance from UV-Vis extinction spectra of *C. sakazakii* incubated with Au NP functionalized with 100% PEG-biotin, streptavidin and anti-C. sakazakii, shown in Figure 4A, after bacteria background subtraction.

References

1. Laemmli, U.K. Cleavage of structural proteins during the assembly of the head of bacteriophage t4. *Nature* **1970**, *227*, 680.

2. Park, Y.-K.; Park, S. Directing close-packing of midnanosized gold nanoparticles at a water/hexane interface. *Chemistry of Materials* **2008**, *20*, 2388–2393.
3. Lundgren, A.; Agnarsson, B.; Zirbs, R.; Zhdanov, V.P.; Reimhult, E.; Höök, F. Nonspecific colloidal-type interaction explains size-dependent specific binding of membrane-targeted nanoparticles. *ACS Nano* **2016**, *10*, 9974–9982.

© 2018 by the authors. Submitted for possible open access publication under the terms and conditions of the Creative Commons Attribution (CC BY) license (http://creativecommons.org/licenses/by/4.0/).
